# Pneumoperitoneum as an uncommon complication after an axillary laceration in a horse

**DOI:** 10.1002/vms3.718

**Published:** 2022-01-06

**Authors:** Linda Marie Schoen, Mohammed Al Naem, Michael Röcken, Florian Geburek

**Affiliations:** ^1^ Department for Equine Surgery Justus‐Liebig Universität Gießen Gießen Germany

**Keywords:** axillary laceration, horse, pneumomediastinum, pneumoperitoneum, pneumothorax

## Abstract

Lacerations of the axillary region occur frequently in horses. Typical complications caused by entrapment of air in the wound during locomotion are subcutaneous emphysema, with consecutive pneumomediastinum and pneumothorax. In this case report, the clinical, radiographic and laboratory diagnosis and management of these complications after an axillary laceration that finally resulted in pneumoperitoneum are described. A 1‐year‐old Hannoveranian was presented with a pre‐existing axillary laceration of unknown duration and subcutaneous emphysema in the surrounding tissue. Due to extensive tissue loss, attempts to adequately close the wound surgically and by packing with sterile dressing material were unsuccessful. Despite stall confinement and tying of the horse, subcutaneous emphysema was progressive and pneumomediastinum as well as pneumothorax was developed. These complications were monitored radiographically. On day 5 after admission, signs of air accumulation were detected on radiographs craniodorsally in the peritoneum and a pneumoperitoneum was diagnosed. Repeated thoracentesis with a teat cannula to gradually evacuate the thoracic cavity was used in combination with nasal oxygen insufflation to treat global respiratory insufficiency. Subcutaneous emphysema and all other complications resolved progressively and the horse was discharged from the hospital 21 days after admission when the axillary wound was adequately filled with granulation tissue. The wound healed fully 1 month later and the horse did not develop long‐term complications within the following year. To the authors´ knowledge, the development of pneumoperitoneum including its radiographic monitoring following an axillary laceration has not been described in horses previously.

## Graphical abstract

Pneumoperitoneum, pneumothorax, and pneumomediastinum resulting from an axillary laceration in a horse.

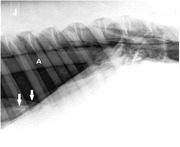

## 1 INTRODUCTION

Axillary wounds are common in horses and are caused by traumatic insults such as running into stationary objects, being impaled by sharp objects or kick injuries from other horses (Joswig & Hardy, 2013). Depending on the size and depth of the wound, air might be entrapped during movement of the limb and migrate along fascial planes and lead to subcutaneous emphysema, pneumomediastinum, and pneumothorax, which may become life‐threatening (Boy & Sweeney, 2000; Hance & Robertson, 1992; Hassel, 2007; Joswig & Hardy, 2013; Mochal et al., 2009).

Pneumoperitoneum is defined as the presence of air in the abdominal cavity. In humans, potential causes of pneumoperitoneum include ruptured hollow viscus, pneumomediastinum, pneumothorax as a result of tracheostomy tube exchange, recent abdominal surgery, leaking surgical anastomosis and misplaced thoracostomy tubes (Agut et al. 2015; Mann et al., 2010; Saeed et al., 2017). Pneumomediastinum, pneumoperitoneum and subcutaneous emphysema secondary to bilateral pneumothorax have been described as a complication after thoracoscopic stabilisation of an anterior spine fracture in humans (Garcia et al., 2009). Other thorax associated non‐surgical causes of pneumoperitoneum in people are barotraumas, asthma, blunt trauma, increased intrathoracic pressure and intermittent positive‐pressure ventilation (Karaman et al., 2005). Animal studies in cats have shown that ventilation with pressure above 60 cm H_2_O leads to the development of subcutaneous emphysema and pneumoperitoneum (Grosfeld et al., 1971).

In cats, pneumoperitoneum has been reported to occur secondary to metastatic pulmonary carcinoma (Greci et al., 2015), while it may occur secondary to blunt force trauma to the chest in dogs (Simmonds et al., 2011). In dogs and cats, trauma‐induced (36%) and spontaneous (64%) pneumoperitoneum, caused by perforation of hollow viscus and pneumothorax, are the most common causes of pneumoperitoneum (Saunders & Tobias, 2003).

Pneumoperitoneum in horses has been described after rupture or perforation in the intra‐abdominal viscus (Pratt et al., 2003).

The aim of this case report is to describe the consecutive development of subcutaneous emphysema, pneumomediastinum, pneumothorax and pneumoperitoneum as complications of an axillary wound in a horse. To the authors' knowledge, pneumoperitoneum has not been reported as a potential consequence of axillary wounds in horses.

## 2 CASE REPORT

### 2.1 Case history

A 1‐year‐old Hannoveranian stallion was referred to the clinic for evaluation and management of a laceration of the right axillary region. The exact age of the wound could not be determined. Neither diagnostic nor therapeutic steps had been undertaken before referral.

### 2.2 Clinical findings

On admission, the heart rate was 44 beats/min, respiratory rate was 12 breaths/min, the rectal temperature was increased at 39.2°C and mucous membranes were pink with a capillary refill time of less than 2 seconds. Auscultation of the thorax revealed normal airway sounds in both lung fields.

During the physical examination, a 10 cm × 4 cm laceration of the right axillary region with skin loss between the region of the *M. pectoralis* and *M. subclavius*, and a diffuse subcutaneous emphysema extending over the right and left thoracic walls were observed. The exact depth of the wound was not definable. Nevertheless, by palpation, the wound depth was estimated to be approximately 3 cm. The wound showed a small amount of serosanguinous exudate. The wound edges were swollen, partly dried superficially and detached from the subcutaneous tissue. The cause of the wound could not be determined. Wound edges and the wound itself were very irregular. There was no granulation tissue. Palpation of the thoracic wall was not indicative of rib fracture. Both forelimbs showed several superficial skin abrasions of the metacarpal region. Initial orthopaedic examination revealed a moderate right forelimb lameness at a walk with shortening of the cranial phase of the stride and moderate swelling of the right metacarpophalangeal joint with severe pain on flexion. Radiographs of the right elbow region (mediolateral and craniocaudal projection) and metacarpophalangeal joint did not show abnormalities of the skeleton.

#### 2.2.1 Laboratory findings

Blood analysis revealed a packed cellular volume of 34 L/l (reference range [rr] 30–43 L/l), total plasma protein 75 g/l (rr 57–72 g/l) white blood cell count 11.89 G/l (rr 5.5–12.5 G/l) with 68.7% neutrophils (rr 45–70%), 26% lymphocytes (rr 20–45%) and 5.1% monocytes (rr 0–5%). Results of arterial blood gas analysis were within normal ranges [pH 7.37 (rr 7.4 ± 0.2), PaCO_2_ 41 mm Hg (rr 40 ± 3), PaO_2_ 91 mm Hg (rr 94 ± 3)].

#### 2.2.2 Diagnostic imaging

**FIGURE 1:**
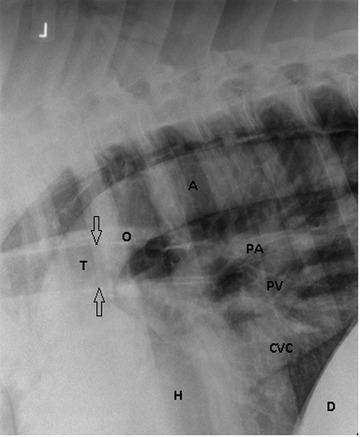
Latero‐lateral radiograph of the craniodorsal thorax; cranial is to the left, image on admission. Due to the presence of air in the mediastinum the outlines of the mediastinal contents such as the oesophagus (O), major vessels (A) aorta, (PA) pulmonary arteries, (PV) pulmonary veins, (CVC) caudal vena cava‐cardiac outlines (black arrows) and outlines of the trachea (T) are abnormally well visualised. A pneumomediastinum was diagnosed. (H) heart, (D) diaphragm

A latero‐lateral radiograph of the thorax taken from the right side showed typical signs of pneumomediastinum (Figure 1). Due to the presence of diffuse subcutaneous emphysema, an ultrasonographic examination did not yield an adequate image quality. During endoscopy of the trachea and oesophagus, no abnormalities were identified.

### 2.3 Treatment

The abrasion wounds on both forelimbs were cleaned and treated locally with povidone‐iodine (Vet‐Sept^®^, aniMed GmbH, Germany) and the limbs were bandaged, in order to cover the wounds and avoid phlegmonous swelling. The horse received tetanus toxoid (10.000 IE i.m.) flunixin‐meglumine (1.1 mg/kg bwt SID first day i.v., then p.o. for 3 days) and cefquinome (1 mg/kg bwt SID first day i.v., then i.m. for 5 days). Fur in the area of the wound edges was clipped. The axillary laceration was debrided and thoroughly lavaged with sterile saline solution. After infiltration of local anaesthetics (Lidocainhydrochlorid 2%) in the wound edges, the skin was adapted with interrupted vertical mattress sutures using multifilament coated polyester (Ethibond^®^ 1 USP). Due to skin loss, a complete closure of the wound was not possible. Thus, it was packed and covered with sterile gauze and sealed with the self‐adhering‐incision drapes (Ioban™ 2) to prevent entrapment of air in the wound. The animal was tied in a stall to minimise movement and was closely monitored.

### 2.4 Progression

**FIGURE 2:**
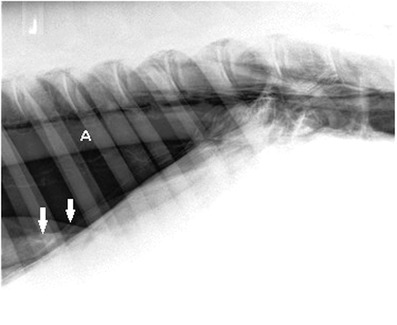
Left latero‐lateral image of the caudodorsal thorax, cranial is to the left, image from day 5. The caudal lung lobes are radiopaque and markedly retracted from the dorsal aspect the pleural cavity. Dorsal to the collapsed lung lobes (border delineated by arrows) the pleural cavity is filled with free air creating a radiolucent area

**FIGURE 3:**
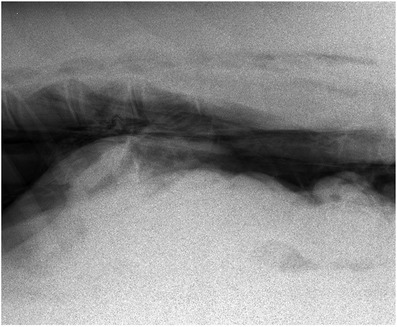
Left latero‐lateral image of the craniodorsal abdomen, cranial is to the left, image from day 5. Air enclosed in the abdominal cavity creates a radiolucency dorsal to radiopaque and irregularly contoured intestine and ventral to the vertebral column

Within 12 hours of hospitalisation, the rectal temperature of the horse decreased to 37.8°C and remained within normal limits. From the second day of admission onward, the axillary wound produced a serosanguinous exudate. The wound was examined and cleaned daily. After changing the sterile gauze, the wound was covered with self‐adhering‐incision drapes (Ioban™ 2). The subcutaneous emphysema increased continuously to the dorsal and ventral aspect of the thorax up to the 12th rib. Additionally, both sides of the neck and head began to show subcutaneous emphysema upon palpation. On the fifth day, a significant rise of the respiratory rate to 44–52 breaths/min and a shallow respiratory pattern were noticed. An arterial blood gas analysis revealed a hypoxemia with PaO_2_ of 69.3 mm Hg, PaCO_2_ of 41.4 mm Hg and a normal pH (7.404). A global respiratory insufficiency was diagnosed. The respiratory distress and the continuously increased subcutaneous emphysema gave reason to repeat radiography of the thorax. While the previously described signs of pneumomediastinum were still evident, radiographic signs were typical of bilateral pneumothorax (Figure 2). Additionally, peritoneal air pockets were suspected in the dorsal abdominal cavity (Figure 3). A pneumoperitoneum was diagnosed.

**FIGURE 4:**
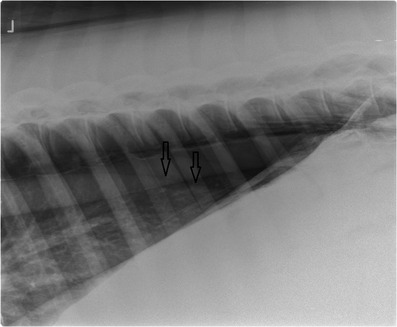
Left latero‐lateral radiograph of the caudodorsal thorax; cranial is to the left, image from day 7. Arrows outline the caudodorsal margins of the left and right collapsed lung lobes consistent with bilateral pneumothorax. The triangular radiolucent area in the caudodorsal pleural cavity is smaller than in the radiograph shown in Figure 2

Nasal insufflation of oxygen at a flow rate of 15 L/min was initiated. Bilateral thoracentesis was performed after local analgesia by inserting a teat cannula in the 14th intercostal space just below the epaxial muscles. A three‐way stopcock was attached to the teat cannula and the cannula was inserted through the skin into the pleural space. Air was evacuated from the thorax using a 100 ml syringe until the horse showed no respiratory distress anymore, and the respiratory rate decreased to 16 breaths/min. Successful evacuation of the thorax was controlled by repeated radiographs. Two days later, radiographs were taken due to a renewed increase of the respiratory rate to 28 breaths/min. Again, signs of pneumothorax, albeit less extended than previously (Figure 4) and pneumoperitoneum were found, and thoracentesis including evacuation of the pleural cavity was repeated. Afterward, the respiratory rate stayed within normal values (12–20 breaths/min). Subcutaneous emphysema receded.

The part of the axillary wound that could not be closed by sutures started to fill with granulation tissue. The sutured part of the wound had healed by the second intention and on day 12 after admission, the sutures were removed from the skin.

### 2.5 Outcome

**FIGURE 5:**
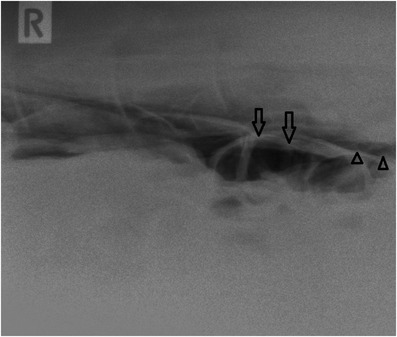
Right latero‐lateral radiograph of the craniodorsal area of the abdomen, image from day of discharge (day 21). Indicating gas‐filled large intestinal loops (arrows) surrounded by small amount of free air in the abdominal cavity (arrowheads)

After 21 days of hospitalisation, the horse was discharged from the clinic. At that time, low‐grade signs of pneumoperitoneum were still evident on radiographs of the cranial abdomen (Figure 5). At the time of discharge, the horse showed no lameness at a walk, the wound in the axillary region was fully covered by granulation tissue. Results from arterial blood gas analysis were within normal ranges. Wound management was continued by the referring veterinarian. Controlled walk on the hand was advised until complete healing of the wound.

Follow‐up information was obtained by telephone interview with the referring veterinarian. One month after discharge the wound had fully healed and was covered with epithelium. A telephone interview with the owner 1 year after discharge from the hospital revealed neither impairment of gait or respiratory function nor other abnormalities. The owner was able to start training the horse.

## 3 DISCUSSION

This report describes the clinical signs and diagnostic findings of a horse with pneumomediastinum, pneumothorax and pneumoperitoneum consecutively resulting from an axillary wound.

In the current case, the horse developed subcutaneous emphysema, pneumomediastinum and pneumothorax after an axillary wound, which are common complications following this type of laceration (Joswig & Hardy, 2013). The axillary wound was treated by packing with gauze, sutures and sealing in combination with restriction of movement as recommended (Barber, 2016; Hendrickson, 2012; Joswig & Hardy, 2013). Although complete closure of the wound by sutures was attempted, it was unsuccessful due to skin loss.

Due to the rapidly developing subcutaneous emphysema with consecutive pneumomediastinum, colonisation and infection with a mixed flora consisting of aerobe and anaerobe bacteria were expected after the initial examination. This was further supported by the failure of primary closure of the axillary wound and finally the development of a pneumoperitoneum. A rapidly acting, well‐tolerated broad‐spectrum bactericidal antibiotic applicable without the need to implant a venous catheter was deemed necessary. Therefore, cefquinome was used in this clinical case.

The complications of axillary lacerations already described in the literature and mentioned above occurred in the present case. This is most likely the result of the failure of airtight closure of the wound. In the current case, the configuration and location of the wound potentially impaired the airtight closure of the wound. Due to the anatomical situation at the elbow region and the incomplete immobilisation of that area, placement of the self‐adhering‐incision drapes was difficult and it had to be changed daily. Furthermore, the exudate of the wound compromised the function of the drapes.

It is likely that air continued to enter the tissues through the wound until the subsidence of subcutaneous emphysema on day 7, and thus the complications of pneumomediastinum and pneumothorax that occurred necessitated further treatment steps. To reduce the amount of subcutaneous emphysema, it has been proposed to incise the skin lateral to the withers to allow air to escape from subcutaneous tissue (Joswig & Hardy, 2013), which, however, may impair self‐limitation of pneumothorax and pneumoperitoneum and implies an increased risk of further complication, for example cellulitis.

The use of an active suction drainage system might have been a more appropriate alternative treatment option. However, due to the skin loss application of, for example, a fenestrated drain (Redon– or Jackson–Pratt Drain) connected to a bellow to create negative pressure did not seem to be rewarding. Application of a vacuum‐assisted closure system, which involves the delivery of intermittent or continuous sub‐atmospheric pressure through a specialised pump connected to a resilient open‐celled foam surface wound dressing covered with an adhesive drape to maintain a closed environment, could have been an alternative (Rijkenhuizen et al. 2005). Unfortunately, this system was not available in the clinic at that time.

In this clinical case, diffuse subcutaneous emphysema and pneumomediastinum were evident on admission to the clinic. Subcutaneous emphysema typically develops slowly to a clinically significant extent, within 2–4 days after injury of the axillary region, as the wound acts as a one‐way valve during locomotion, leading to a gradually increasing volume of air being entrapped within subcutaneous tissue (Joswig & Hardy, 2013). Since subcutaneous emphysema and pneumomediastinum were present on admission, it is assumed that the wound developed several days before presentation in the hospital.

The development of pneumomediastinum and pneumothorax after axillary lacerations has been described as a result of air entering the subcutaneous tissue, migrating along the fascial planes in the mediastinum, causing pneumomediastinum and consequently tension pneumothorax, as the two pleural cavities communicate with each other, via fenestrations in the caudal and ventral mediastinum in horses (Nickel et al., 1979). One study on thoracoscopy in horses showed a higher risk for the development of bilateral pneumothorax after right‐sided thoracoscopy than after left‐sided thoracoscopy (Scharner, 2012). A right‐sided valve‐function of the mediastinal fenestrations was considered as a potential reason. This theory remains hypothetical and requires further anatomical studies on the mediastinal fenestrations in horses.

The exact pathomechanism of the pneumoperitoneum in the current case is not clear. In humans, pneumomediastinum and pneumothorax can lead to pneumoperitoneum as the spaces between subcutaneous tissue, the two sheets of mediastinum and retroperitoneum are anatomically continuous (Sato et al., 2011), so that air may pass via the mediastinum, along the major diaphragmatic openings, such as the aortic and oesophageal hiatus to the retroperitoneum and further into the peritoneal space (Karaman et al., 2005). In the present case, air may have entered the peritoneal cavity indirectly, as described in humans. However, an anatomical study in equids may be required to confirm this hypothesis.

Although other potential reasons for pneumoperitoneum, for example, concurrent laceration of abdominal hollow viscus were considered, they could be excluded by the progression of the case. Although very unlikely, a laceration of the diaphragm due to trauma in the context of the axillary wound, as well as a congenital or acquired diaphragmatic defect, could have promoted pneumoperitoneum, as a consequence of pneumothorax and pneumomediastinum. Also, direct penetration of the abdomen was excluded, due to the extent of the wound and the anatomic localisation. The wound at the elbow region was at least 25 cm distant from the most ventrocranial part of the abdominal cavity.

Due to the generalised subcutaneous emphysema ultrasonography of the thorax did not yield adequate image quality. In humans, the most common diagnostic modalities for detecting pneumothorax and pneumoperitoneum are ultrasound and radiography (Boy & Sweeney, 2000; Kim et al., 2014). An experimental study by Partlow et al. (2017) found that ultrasound (2D and M‐mode) is more sensitive than radiography for the detection of small volume induced‐pneumothorax in horses.

Pneumomediastinum was diagnosed, radiographically, in the current case. This modality has been referred to as the primary modality to identify air in the mediastinum and the pleural cavity (Prange, 2015). Ultrasound is not adequate to visualise the mediastinum, due to the surrounding aerated lungs (Prange, 2015).

In our case, pneumoperitoneum could be successfully diagnosed using radiography. In humans, ultrasonography has good accuracy and reliability in the detection of pneumoperitoneum (Nazerian et al., 2015). In dogs, the minimum amount of abdominal free gas that could be identified by ultrasonography for diagnostic purposes was 0.2 ml (Kim et al., 2014). Abdominal ultrasound is best performed while the animal is in dorsal recumbency (Kim et al., 2014). However, in horses, abdominal ultrasonography is usually done while the animal is in a standing position, making the detection of a small amount of free air more difficult.

In the reported case, pneumoperitoneum was still evident radiographically 21 days after it had developed. As the horse could not be further reevaluated in our clinic, the exact time until full resolution of pneumoperitoneum remained unclear. While in dogs, the radiographic resolution of pneumoperitoneum varies between 9 and 25 days and depends on the volume of air in the peritoneal space (Agut et al., 2015), respective data for horses are not available. In our case, pneumoperitoneum developed secondary to an axillary laceration with consecutive pneumothorax and was regressive after treatment of these conditions, so that a targeted treatment of pneumoperitoneum was not necessary. The treatment of pneumoperitoneum consists primarily in the elimination of the cause free abdominal gas will then be absorbed by the organism. Only in the rare complication of a tension pneumoperitoneum, the placement of an abdominal drain is described (Hillman 1983).

## 4 CONCLUSIONS

The present study reports a case of subcutaneous emphysema, pneumomediastinum, pneumothorax and pneumoperitoneum following an axillary laceration. In conclusion, management of an axillary wound with extensive skin loss, by sealing and packing it and restriction of the movement might not be sufficient to prevent the development of pneumoperitoneum, which has to be considered as a potential complication after this type of laceration. Radiography is the diagnostic modality of choice to detect pneumoperitoneum in horses.

## CONFLICT OF INTEREST

The authors declare no conflict of interest.

## AUTHOR CONTRIBUTIONS

**Linda Schoen** was associated with conceptualisation, investigation, and wrote the original draft. **Mohammed Al Naem** was associated with conceptualisation, investigation, and reviewed and edited the final manuscript. **Michael Röcken** reviewed and edited the final manuscript. **Florian Geburek** was associated with conceptualisation, investigation; methodology, and reviewed and edited the final manuscript. [Correction added on 12 January 2022, after first online publication: The second sentence was corrected]

### PEER REVIEW

The peer review history for this article is available at https://publons.com/publon/10.1002/vms3.718

## Data Availability

The authors confirm that the main data supporting the findings of this case report are presented within the article. Further supporting data are available from the corresponding author, i.e. Dr. Linda Schön [lindaschoen@gmx.de], upon reasonable request.
